# Comprehensive Investigation of Ginsenosides in the Steamed *Panax quinquefolius* with Different Processing Conditions Using LC-MS

**DOI:** 10.3390/molecules29030623

**Published:** 2024-01-28

**Authors:** Jiali Fan, Feng Liu, Wenhua Ji, Xiao Wang, Lili Li

**Affiliations:** 1 Key Laboratory for Applied Technology of Sophisticated Analytical Instruments of Shandong Province, Shandong Analysis and Test Center, Qilu University of Technology (Shandong Academy of Sciences), Jinan 250014, China; 17731638714@163.com (J.F.); liufeng8109@163.com (F.L.); jwh519@163.com (W.J.); wangx@sdas.org (X.W.); 2 School of Pharmaceutical Sciences, Qilu University of Technology (Shandong Academy of Sciences), Jinan 250014, China

**Keywords:** steamed *Panax quinquefolius*, ginsenosides, identification, processing, LC-MS

## Abstract

*Panax quinquefolius* (PQ) has been widely used in traditional Chinese medicine and functional food. Ginsenosides are the important functional components of PQ. The ginsenosides’ diversity is deeply affected by the processing conditions. The ginsenosides in the steamed PQ have been not well-characterized yet because of the complexity of their structure. In the study, the comprehensive investigation of ginsenosides was performed on the steamed PQ with different steaming times and temperatures by UPLC-Q-TOF-MS. Based on the molecular weight, retention time and characterized fragment ions, 175 ginsenosides were unambiguously identified or tentatively characterized, including 45 protopanaxatriol type, 49 protopanaxadiol type, 19 octillol type, 6 oleanolic acid type ginsenosides, and 56 other ginsenosides. Ten new ginsenosides and three new aglycones were discovered in the steamed PQ samples through searching the database of CAS SciFinder^n^. Principal component analysis showed the significant influence on the chemical components of PQ through different processing conditions. The steaming temperature was found to promote the transformation of ginsenosides more than the steaming time. The protoginsenosides were found to transform into the rare ginsenosides by elimination reactions. The malonyl ginsenosides were degraded into acetyl ginsenosides, and then degraded into neutral ginsenosides. The sugar chain experienced degradation, with position changes and configuration inversions. Furthermore, 20 (*S*/*R*)-ginsenoside Rh1, Rh2, Rg2, and Rh12 were found to transform from the S-configuration to the R-configuration significantly. This study could present a comprehensive ginsenosides profile of PQ with different steaming conditions, and provide technical support for the development and utilization of PQ.

## 1. Introduction

*Panax quinquefolius* (PQ) belongs to the genus *Panax* in the family Araliaceae. It has been widely used in traditional Chinese medicine, dietary supplements, and functional food [1,2]. It is native to the south of Canada and the northern USA, and has been widely planted in China. In vivo and in vitro studies have shown that PQ has many biological activities, including antioxidative, antidiabetic, anti-inflammatory, anti-cancer, etc., [3,4]. It has been demonstrated to have a positive impact on the treatment of various diseases such as in the central nervous system, endocrine system, cardiovascular system, as well as cancer [5]. In particular, PQ has unique advantages in treating chronic diseases because of its better compatibility with the human body and fewer side effects [3]. It was found that steamed PQ has been reported to exhibit enhanced antiproliferative activity and antioxidant capacity [6,7]. PQ contains many chemical components, including ginsenosides, polyacetylenes, polyphenolic compounds, etc. Ginsenosides are the most important active ingredients [8,9]. More than 500 ginsenosides have been discovered from *Panax* species [10], but only a few have been characterized in the steamed PQ samples. A comprehensive investigation of ginsenosides is essential for the further development and utilization of the steamed PQ.

Based on the structure difference of the aglycone, ginsenosides are divided into protopanaxadiol (PPD-type), protopanaxatriol (PPT-type), octillol (OT-type), oleanolic (OA-type), and other types [11]. OT-type ginsenosides are characteristic in the PQ, differing from other *Panax* species [12]. The ginsenosides Rb1, Rb2, Rc, Rd, Re, and Rg1 are generally considered as the main protoginsenosides. Protoginsenosides have a larger molecular structure that is difficult to be absorbed by the human body, whereas less-polar ginsenosides are easily absorbed by the intestinal microflora [13,14]. These ginsenosides are called rare ginsenosides, and they have been found to have special pharmacological activities [15]. Compared to ginsenoside Rb1, ginsenoside Rg5 has been demonstrated to have stronger antiproliferative activity against breast cancer [16,17]. Ginsenoside Rh4 has been reported to have anti-esophageal cancer effects through inhibiting aerobic glycolysis [18,19]. Rare ginsenosides can be prepared by physical, chemical, and biological methods [20]. The chemical method has usually the disadvantage of a long reaction time and the production of byproducts. The biotransformation of rare ginsenosides needs to solve the problems of low specific activities, unidentified enzymes, and uncovered catalytic mechanisms [21]. The physical method commonly refers to the steaming method. It is a green and efficient method to regulate the transformation of ginsenosides [22]. Ginsenosides undergo chemical modifications and generate rare ginsenosides during the steaming process [23]. There have been several reports on steamed PQ. Twelve ginsenosides were determined in the roots of steamed PQ, and the anticancer activities of the extract from roots steamed for 2 h were greater than 1 h [24]. Twenty-nine major ginsenosides have been studied in the multi-steamed PQ samples and a possible chemical conversion was deduced [25]. Fifty-nine ginsenosides of PPT, PPD, OA, and OT types have been analyzed in PQ with different steaming processes [26]. However, a few known ginsenosides cannot meet the needs of holistic studies or the discovery of chemical markers in the PQ steaming process [27]. Further studies of the steam processing mechanism will be of great significance for the rational utilization of PQ.

The development of analytical technologies has made it possible for an holistic study. LC-MS has been widely used in secondary metabolite analysis for its high sensitivity and high throughput, such as for flavonoids, phenolic acids, and ginsenosides [28,29]. In the study, ginsenosides profiling has been established for the steamed PQ samples based on LC-MS. The in-depth identification of PQ was performed, the aglycone and the sugar chains were annotated, and new ginsenosides were discovered. The structure and content changes of the ginsenosides were analyzed with the different steaming processes, and the transformation mechanisms of ginsenosides were further investigated. The study could present the comprehensive ginsenoside profiling of steamed PQ with different times and temperatures.

## 2. Results and Discussion

### 2.1. Identification of Ginsenosides in PQ Samples

**Figure 1:**
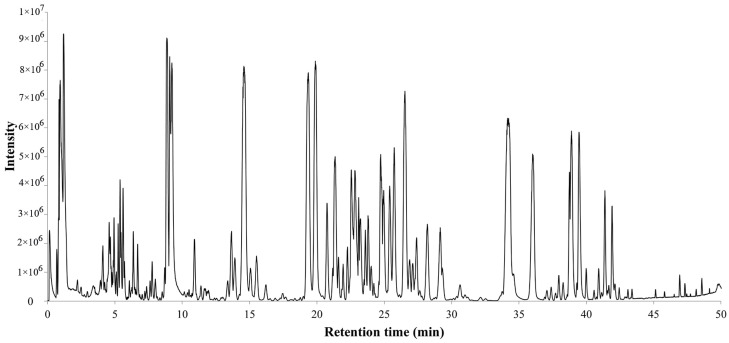
Total ion chromatogram of steamed PQ with LC-MS in the negative ion mode.

**Figure 2:**
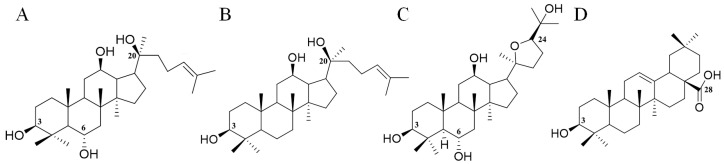
The chemical structures of PPT- (**A**), PPD- (**B**), OT- (**C**), and OA- (**D**) type aglycones. Numbers represent the typical glycosylation sites.

The total ion chromatogram of the PQ sample with LC-MS is shown in Figure 1. For ginsenosides, there were specific fragmentation rules in the MS/MS analysis [30]. The identification of ginsenosides was based on the accurate molecular weight, retention time, and MS/MS fragment ions. With different collision energies in the MS/MS analysis, ginsenosides were fragmented into aglycone and sugar chains. Characterized aglycone ions, sugar chain ions, and the neutral loss of sugar chains were found. For the PPT-, PPD-, OT-, or OA-type, the characterized aglycone ions were 475, 459, 491, and 455 (*m*/*z*), respectively. The chemical structures of the PPT-, PPD-, OT-, or OA-type aglycones are shown in Figure 2. The aglycone ions of other types of ginsenosides were complicated and varied. The characteristic ions for different sugar chains of *O*-glucose, *O*-rhamnose, *O*-xylose/arabinose, *O*-glucose-glucose, *O*-glucose-rhamnose, and *O*-glucose-xylose/arabinose were 161, 145, 131, 221/323, 205/307, and 191/293, respectively. In addition, acetyl and malonyl ginsenosides were characterized as having a neutral loss of 42 and 86. Then, a total of 175 ginsenosides were identified from the extracts of the PQ samples, which included 45 PPT-type, 49 PPD-type, 19 OT-type, 6 OA-type ginsenosides, and 56 other ginsenosides. The ginsenosides were then validated with the standards available. The detailed molecular weight, retention time, aglycone, sugar chains, and MS/MS fragment ions of the ginsenosides are listed in Table 1.

**Figure 3:**
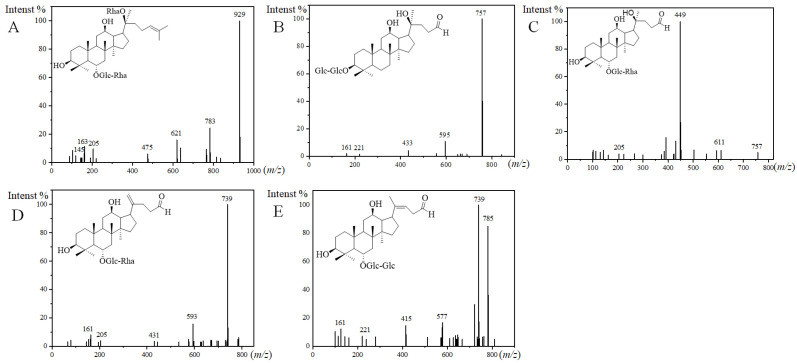
MS^2^ spectra and the presumed structures of PPT-*O*-glc-rha/*O*-rha ((**A**), 50 eV), PQ-ginsenoside A ((**B**), 40 eV), PQ-ginsenoside B ((**C**), 70 eV), PQ-ginsenoside C ((**D**), 40 eV), and PQ-ginsenoside D ((**E**), 40 eV).

For the structures of the ginsenosides, the database CAS SciFinder^n^ was searched. Among them, 10 ginsenosides were identified in the steamed PQ for the first time. The aglycone or sugar chains were different compared with the ginsenosides reported previously. There was one new ginsenoside classified as the PPT-type, and it was named PPT-*O*-glc-rha/*O*-rha. PPT-*O*-glc-rha/*O*-rha (*m*/*z* 975.5534, [M+HCOO]^−^) was identified by fragment ions of 929, 783, 621, 475, 205, 163, 161, and 145 (Figure 3A). The fragment ion of 475 is the characteristic aglycone ion of PPT-type ginsenosides. The neutral loss of 929/783, 783/621, and 621/475 indicated two rhamnose and a glucose in the sugar chains. The fragment ion of 205 indicated a sugar chain of *O*-glucose-rhamnose.

There were nine new ginsenosides classified as the other type, and they were named PQ-ginsenoside A, B, C, D, acetyl-PQ-ginsenoside A, and acetyl-PQ-ginsenoside D isomers. Three new aglycones were discovered in PQ-ginsenoside B, C, and D, and the *m*/*z* of them were 449, 431, and 415 in the MS/MS analysis. For example, the fragmentation rules of PQ-ginsenoside C were consistent with the general ginsenosides, and a new aglycone with *m*/*z* 431 was discovered. The *m*/*z* of the aglycone in PQ-ginsenoside A was 433. The aglycone with *m*/*z* 433 has been reported to be degraded from 459 in the PPD-type, and it was characterized as 25-, 26-, and 27-trinor-PPD-type in the floralginsenoside Kb from *Panax* ginseng [31,32]. The sugar chain of PQ-ginsenoside A was different from floralginsenoside Kb, and it was not found in the database of CAS SciFinder^n^, so it was defined as a new ginsenoside. The sugar chains of PQ-ginsenoside A were the same as Rg3, and they were eluted at the same retention time. Rg3 is a PPD-type ginsenoside. Therefore, the aglycone structure in PQ-ginsenoside A were deduced as 25-, 26-, and 27-trinor-PPD-type degraded from Rg3 (Figure 3B). For PQ-ginsenoside B (aglycone, *m*/*z*, 449, Figure 3C), it has the same sugar chains and retention time with Rg2 (aglycone, *m*/*z*, 475). For PQ-ginsenoside C (aglycone, *m*/*z*, 431, Figure 3D), it has the same sugar chains and retention time with Rg6 (aglycone, *m*/*z*, 457). For PQ-ginsenoside D (aglycone, *m*/*z*, 415, Figure 3E) it has the same sugar chains and retention time with Rg5 (aglycone, *m*/*z*, 441). The molecular weight difference of 449/475, 431/457, and 415/441 were the same as the 433/459. Therefore, the aglycone structures of 449, 431, and 415 in PQ-ginsenoside B, C, and D were deduced to be degraded from 475, 457, and 441 in the Rg5, Rg6, and Rg5, characterized as 25-, 26-, and 27-trinor.

**Figure 4:**
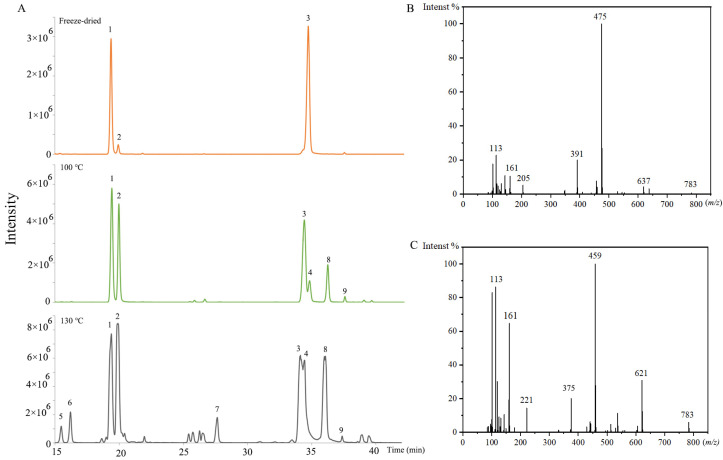
(**A**) The extracted ion chromatogram of 829.4943 (*m*/*z*) in the freeze-dried sample, and steamed samples (100 °C, 2 h), and (130 °C, 2 h), No. 1, 2, 5, and 6 represent the ginsenoside Rg2 and its isomers, No. 3, 4, 7, 8, and 9 represent the ginsenoside Rg3 and its isomers. (**B**) MS^2^ spectrum of 20(*S*)-Ginsenoside Rg2. (**C**) MS^2^ spectrum of 20(*S*)-Ginsenoside Rg3 (70 eV).

There were also many isomers identified. For the isomers, the fragmentation ions in the MS/MS analysis were the same. The aglycone and sugar chains of them were the same as the previous ginsenosides, but the attaching positions of the sugar chain and aglycone were different. The extracted ion chromatograms of 829.4943 (*m*/*z*) in the freeze-dried sample, the (100 °C, 2 h) sample, and the (130 °C, 2 h) sample are shown in Figure 4A. It was obvious that the ginsenosides contents and varieties were changed with the increase in steaming temperature. The ginsenoside Rg2 and Rg3 were PPT-type and PPD-type, respectively. The ginsenoside Rg2 isomers (No. 1, 2, 5, and 6) were identified by product ions of 783, 637, 475, 205, 161, and 145 (Figure 4B). The ginsenoside Rg3 isomers (No. 3, 4, 7, 8, and 9) were identified by product ions of 783, 621, 459, 221, and 161 (Figure 4C). The No. 1, 2, 3, and 4 were confirmed by standards as 20(*S*)-ginsenoside Rg2, 20(*R*)-ginsenoside Rg2, 20(*S*)-ginsenoside Rg3, and 20(*R*)-ginsenoside Rg3. For PPT-type ginsenosides, the glycosidic bonds were commonly at the aglycone C-6 and C-20 hydroxyl groups [33]. Ginsenoside Rg2 was C-6 linked. Therefore, No. 5 and 6 were deduced as sugar chains linking to the C-20 position, named as the ginsenoside Rg2 isomer. For PPD-type ginsenosides, the glycosidic bonds were commonly at the aglycone C-3 and C-20. Ginsenoside Rg3 was C-3 linked. Therefore, No. 7, 8, and 9 were speculated as sugar-chain-linking to the C-20 position, named the ginsenoside Rg3 isomers.

### 2.2. Method Validation

The repeatability and precision of the analytical method were investigated by QC samples. The repeatability was investigated by six QC samples. The QC samples were analyzed continuously. The RSD of each peak was calculated among the six QC samples. The peak number and area were counted within different RSD ranges (0–10%, 10–20%, 20–30%, and >30%). In total, 98.2% of the peaks had an RSD value of less than 20%, while the accumulated peak area accounted for 99.3% of the total area (Figure S1A). For the intra-day precision analysis, six QC samples were analyzed every 4 h, and 98.1% of the peaks had an RSD less than 20%, while the accumulated peak area accounted for 99.3% of the total peak area (Figure S1B). For the inter-day precision analysis, 18 QC samples were analyzed for 3 days. The results show that 96.0% of the peaks had an RSD of less than 20%, while the accumulated peak area accounted for 98.6% of the total peak area (Figure S1C). These results indicated the good stability of the analytical method.

### 2.3. Difference between Steamed and Freeze-Dried Samples

**Figure 5:**
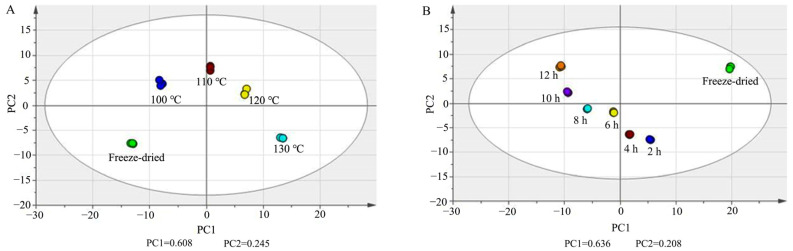
PCA score plots of ginsenosides in PQ samples with different steaming temperatures (**A**) and times (**B**).

Principal component analysis (PCA) was carried out in PQ samples with different steaming times and temperatures with UV scaling. In Figure 5A, PC1 and PC2 were 0.608 and 0.245. In Figure 5B, PC1 and PC2 were 0.636 and 0.208. The score plots show that there was obvious separation among the freeze-dried samples and steamed samples.

A non-parametric test was then performed, and the ratio was calculated between each of the two groups. *p* < 0.05 and ratio > 5 were set as the criteria to screen the differential ginsenosides (Tables S1 and S2). There were 51, 55, 58, 63, 67, and 75 differential ginsenosides found between the steamed samples of 2 h, 4 h, 6 h, 8 h, 10 h, and 12 h and freeze-dried samples, respectively. There were 48, 60, 74, and 89 differential ginsenosides found between steamed samples of 100 °C, 110 °C, 120 °C, and 130 °C and freeze-dried samples, respectively. This indicated that the steam temperature and time had a significant influence on the steamed PQ samples.

### 2.4. Influence of Steam Temperature and Time on the Ginsenosides Composition

**Figure 6:**
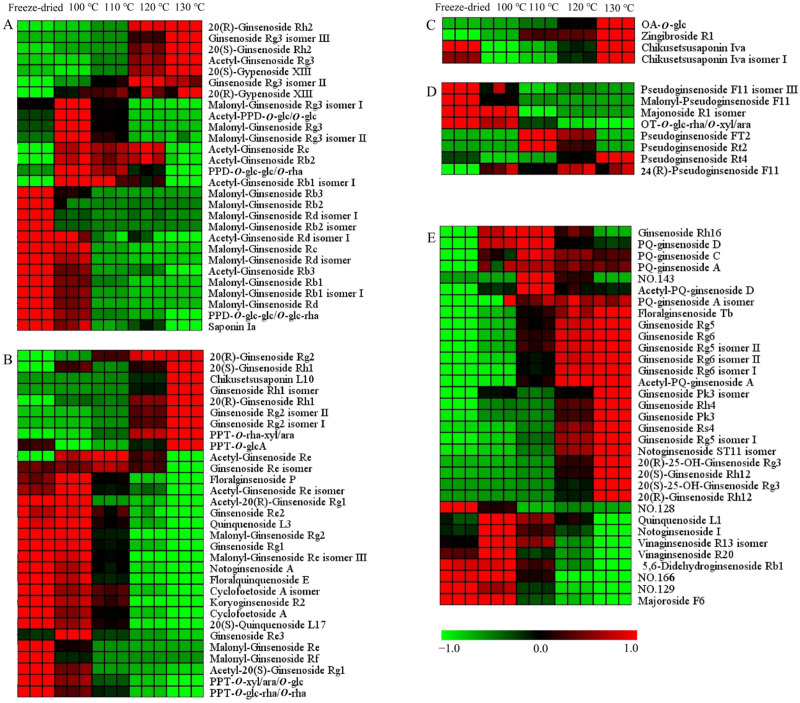
Heat map of differential ginsenosides in PQ samples with different steaming temperatures. (**A**) PPD-type. (**B**) PPT-type. (**C**) OA-type. (**D**) OT-type. (**E**) Other type. No. 128 and 129 represent Dammarane-3,6,12,24,25-pentol, 20-(β-d-glucopyranosyloxy)-, (3β,6β,12β)-(ACI), and isomer. No. 143 represents β-d-Glucopyranoside, (3β,12β)-3,12,24,25-tetrahydroxy-20-(d-xylopyranosyloxy)dammaran-6-yl (ACI). No. 166 represents (3β,12β)-20-(β-d-Glucopyranosyloxy)-3,12,24,25-tetrahydroxydammaran-6-yl 2-*O*-(6-deoxy-α-l-β-d-mannopyranosyl)-β-d-glucopyranoside.

The hierarchical cluster analysis was carried out on the differential ginsenosides. The data were normalized, and the heat map is shown in Figure 6 and Figure S2. There were 104 and 86 differential ginsenosides screened for PQ samples with different steaming temperatures and times, respectively. With the increase in the steaming temperature, the content of 37 ginsenosides increased gradually, while the content of 36 ginsenosides decreased gradually. With the increase in the steaming time, the content of 42 ginsenosides increased gradually, while the content of 15 ginsenosides decreased gradually. In addition, there were some ginsenosides that changed with the steaming time and temperature, for example, climbing up and then declining. The steaming process not only changed the ginsenosides content, but also influenced their structure.

For the PPD-ginsenosides, the difference was shown in Figure 6A and Figure S2A. Firstly, the malonyl-ginsenosides Rb1, Rb2, Rb3, Rd, and Re decreased significantly with the steaming time and temperature. The rate among different samples was calculated. The content of malonyl-ginsenoside Rb3 in the freeze-dried sample was 2-, 323-, and 574-fold more than in the samples (100 °C, 2 h), (100 °C, 12 h), and (130 °C, 2 h), respectively. Malonyl-ginsenoside Rg3 was accumulated after the steaming process, and then decreased with the increase in the steaming time and temperature. Malonyl-ginsenoside Rg3 was increased by 5-fold in the sample (100 °C, 2 h), and then decreased by 26- and 47-fold in the samples (100 °C, 12 h) and (130 °C, 2 h), respectively. Secondly, the acetyl-ginsenosides showed a significant increase after the steaming process. Then, the acetyl-ginsenosides Rc, Rb3, Rb1, and Rd changed slightly with the steaming time, but showed an obviously decline with the increase in the steaming temperature. Acetyl-ginsenoside Rg3 was enhanced from steaming for 8 h at 120 °C. Thirdly, the levels of ginsenosides Rh2, Rg3, and Rb3 and gypenoside XIII were enhanced with the steaming process, while PPD-*O*-glc-glc/*O*-glc-rha and saponin Ia were decreased. And, the steaming time was not significant with the ratios. The content of 20(*R*)-ginsenoside Rh2 was extremely low in the freeze-dried sample, and then increased quickly after the steaming process. Although the content of 20(*S*)-ginsenoside Rh2 was also rising, the increase rate was low. The contents of 20(*R*)-ginsenoside Rh2 and 20(*S*)-ginsenoside Rh2 in the sample (100 °C, 2 h) were 22- and 4-fold more than in the freeze-dried sample, indicating the transformation from the S-configuration to the R-configuration. The contents of 20(*R*)-ginsenoside Rh2 in the samples (100 °C, 12 h) and (130 °C, 2 h) were 8 and 18 times more than in the sample (100 °C, 2 h), respectively. This shows that a high temperature enhances configuration transformation. The malonyl ginsenosides were sensitive to the heat process. The acetyl-ginsenoside could be produced by the decarboxylation of the malonyl group [34]. Therefore, with the decrease in the malonyl ginsenosides, the level of acetyl ginsenosides was enhanced. With the rise in temperature, acetyl ginsenosides were then degraded into neutral ginsenosides. The ginsenoside Rg3 could be produced from acetyl-ginsenoside Rg3, and could be further converted into ginsenoside Rh2 through the elimination of glucose at C-3 [35]. The acetyl-ginsenoside Rg3 could be produced from malonyl-ginsenoside Rg3. The possible transformation pathways are shown in Figure S3.

In the PPT-type (Figure 6B and Figure S2B), the malonyl ginsenosides Re, Rf, and Rg2 decreased with the steaming time and temperature. Acetyl-ginsenoside Re and its isomer showed a rising and then declining tendency, indicating the conversion of malonyl-ginsenosides Re. Configuration transformations were also found in the 20(*S*/*R*)-ginsenoside Rh1 and 20(*S*/*R*)-ginsenoside Rg2. The contents of 20(*S*)-ginsenoside Rh1 and 20(*R*)-ginsenoside Rh1 in the sample (130 °C, 2 h) were 15- and 750-fold more than in the freeze-dried sample, respectively. The contents of 20(*S*)-ginsenoside Rg2 and 20(*R*)-ginsenoside Rg2 in the sample (130 °C, 2 h) were 4- and 59-fold more than in the freeze-dried sample, respectively. PPT-*O*-rha-xyl/ara, chikusetsusaponin L10 and PPT-*O*-glcA were also increased, while ginsenoside Rg1, Re2, quinquenoside L3, PPT-*O*-xyl/ara/*O*-glc, PPT-*O*-glc-rha/*O*-rha, floralginsenoside P, floralquinquenoside E, Cyclofoetoside A, and 20(*S*)-quinquenoside L17 were decreased. The decreased ginsenosides had three or four sugars in the sugar chain, while the increased ginsenosides had one or two sugars. The sugar chains experienced hydrolysis and dehydration. The ginsenoside Re lost the C-6 sugar to generate ginsenoside Rg2 [36]. The PPT-*O*-glc-rha/*O*-rha lost the C-20 sugar to generate ginsenoside Rg2. The ginsenoside Rg2 lost the C-6 sugar to generate Rh1. The possible transformation pathways are shown in Figure S4.

For the OA-type ginsenosides, chikusetsusaponin Iva and isomer I had the same sugar chains (a glucose and a glucuronic acid attached to different positions of the aglycone). They were decreased after the steaming process (Figure 6C and Figure S2C). The content of chikusetsusaponin Iva and isomer I in the freeze-dried sample were 10 and 14 times higher than in the sample (100 °C, 2 h), respectively. Zingibroside R1 was different at the sugar chains in the structure compared with chikusetsusaponin Iva and isomer I. It had a disaccharide chain of glucose and glucuronic acid. OA-*O*-glc had glucose in the sugar chain. The contents of OA-*O*-glc and zingibroside R1 were increased after the steaming process. The contents of OA-*O*-glc and zingibroside R1 in the sample (100 °C, 2 h) were 12 and 6 times more than in the freeze-dried sample. The sugar chain of ginsenoside Ro has been reported to experience degradation to form the zingibroside R1 and chikusetsusaponin Iva [27]. Moreover, it has been deduced that the sugar chain of chikusetsusaponin Iva experiences degradation at C-3 to form OA-*O*-glc. With the increase in the steaming time and temperature, the content of them were also increased. From 2 h to 10 h, the contents of them increased continuously. At the 12 h, their content showed a little decline. The contents of chikusetsusaponin Iva, chikusetsusaponin Iva isomer I, zingibroside R1, and OA-*O*-glc in the sample (100 °C, 10 h) were 4, 3, 4, and 5 times more than in the sample (100 °C, 2 h), respectively. From 100 °C to 130 °C, the contents of them increased continuously. The contents of chikusetsusaponin Iva, chikusetsusaponin Iva isomer I, zingibroside R1, and OA-*O*-glc in the sample (130 °C, 2 h) were 9, 12, 4, and 29 times more than in the sample (100 °C, 2 h), respectively. The steaming temperature had a larger impact on the OA-type ginsenosides’ conversion than the steaming time. The possible transformation pathways are shown in Figure S5A.

OT-type ginsenosides are characteristic ingredients of PQ which different from other *Panax* species [27]. The differences are shown in Figure 6D and Figure S2D. In Figure 6D, Pseudoginsenoside F11 isomer III, malonyl-pseudoginsenoside F11, and OT-*O*-glc-rha/*O*-xyl/ara show a declining tendency, and pseudoginsenoside Rt4, Rt2, Ft2, and 24(*R*)-pseudoginsenoside F11 show a rising tendency. The contents of malonyl-pseudoginsenoside F11 and OT-*O*-glc-rha/*O*-xyl/ara in the sample (100 °C, 2 h) were 69 and 28 times more than in the sample (130 °C, 2 h). The content of malonyl-pseudoginsenoside F11 in the sample (100 °C, 2 h) was 27 times more than in the sample (100 °C, 12 h). The malonyl-pseudoginsenoside F11 and OT-*O*-glc-rha/*O*-xyl/ara underwent demalonylation and hydrolysis reactions to produce the pseudoginsenoside F11, respectively. Pseudoginsenoside Rt4 increased with the time and temperature. The contents of pseudoginsenoside Rt4 in the sample (100 °C, 12 h) and (130 °C, 2 h) was 5 and 10 times more than in the sample (100 °C, 2 h), respectively. The content of 24(*R*)-pseudoginsenoside F11 in the sample (130 °C, 2 h) was six times more than in the freeze-dried sample. The content of pseudoginsenoside Rt2 was the highest at 110 °C, and then declined. The sugar chain of 24(*R*)-pseudoginsenoside F11 (the sugar chain of glucose–rhamnose) and pseudoginsenoside Rt2 (the sugar chain of a glucose–xylose) underwent degradation to form the pseudoginsenoside Rt4 (the sugar chain of a glucose). These results indicate the degradation of the sugar chains with the steaming process, and the high temperature facilitated the degradation process. The possible transformation pathways are shown in Figure S5B.

In Figure 6E and Figure S2E, most other-type ginsenosides increase with the steaming process. Firstly, the steaming time and temperature have little difference in their influence on ginsenoside Rg5, floralginsenoside Tb, ginsenoside Rg6, ginsenoside Rg5 isomer II, and ginsenoside Rg6 isomer II. The contents of ginsenoside Rg5 in the samples (100 °C, 12 h) and (130 °C, 2 h) were five and four times more than in the sample (100 °C, 2 h), respectively. The contents of ginsenoside Rg6 in the samples (100 °C, 12 h) and (130 °C, 2 h) was four- and three-fold higher than in the sample (100 °C, 2 h), respectively. Secondly, the effect of the steaming temperature on the contents of ginsenoside Rs4, ginsenoside Rh4, ginsenoside Rg6 isomer I, 20(*R*)-ginsenoside Rh12, PQ-ginsenoside A, C, D, and acetyl-PQ-ginsenoside D was greater than that of the steaming time. The contents of ginsenoside Rs4 in the sample (100 °C, 12 h) and temperature sample (130 °C, 2 h) was 8 and 40 times that in the sample (100 °C, 2 h). Thirdly, the effect of the steaming time on the contents of ginsenoside Pk3, ginsenoside Rg5 isomer I, and 20(*S*)-ginsenoside Rh12 was greater than that of the steaming temperature. The increased rate of 20(*S*)-ginsenoside Rh12 was less than 20(*R*)-ginsenoside Rh12, indicating the transformation from an S-configuration to an R-configuration. With the steaming process, the protoginsenosides could be transformed into rare ginsenosides. The dehydration of the side chain of C17 resulted in the degradation of ginsenoside Rg3 to ginsenoside Rg5, respectively. Acetyl-ginsenoside Rg3 could be transformed into ginsenoside Rs4 by dehydration in the side chain of C17. Ginsenoside Rg2 could be transformed to ginsenoside Rg6 by dehydration in the side chain of C17. Then, the hydrolysis of the the rhamnosyl residue at C-6 of ginsenoside Rg6 can lead to its degradation into ginsenoside Rh4. The other types ginsenosides were mostly changed in the side chain at C17 of the aglycone (Figures S3 and S4). The ginsenosides underwent a series of chemical reactions, including dehydration, hydrolysis, isomerization, demalonylation, and deacetylation during the steaming process.

## 3. Materials and Methods

### 3.1. Chemical and Reagents

Acetonitrile and methanol were purchased from Merck (HPLC grade, Darmstadt, Germany). Formic acid was purchased from Honeywell (for mass spectrometry, Seelze, Germany). Ultrapure water was produced by a Direct-Q 8 UV-R water purification system (Millipore, Billerica, MA, USA). Ginsenosides Rb1, Ro, Rb2, Rd, Rg1, Rg5, Re, 20(*S*)-ginsenoside Rh1, 20(*S*)-ginsenoside Rg3, 20(*R*)-ginsenoside Rg3, 20(*S*)-ginsenoside Rg2, 20(*R*)-ginsenoside Rg2, 20(*R*)-ginsenoside Rh2, 20(*S*)-ginsenoside Rh2, and 24(*S*)-pseudoginsenoside F11 were purchased from Chengdu Desite Co., Ltd. (Chengdu, China).

### 3.2. Sample Information and Preparation

Four-year-old PQ roots were collected in the plantation farm of Weihai city, Shandong province, China. Fresh PQ roots were steamed at 100 °C for 2 h, 4 h, 6 h, 8 h, 10 h, and 12 h, respectively. And fresh PQ roots were steamed at 100 °C, 110 °C, 120 °C, and 130 °C for 2 h, respectively. Then the steamed PQ roots were cut into 2–3 mm slices and dried at 60 °C. The dried slices were then ground into powder. A batch of fresh roots were cut into 2–3 mm slices and freeze-dried as raw samples. Quality control (QC) samples were prepared by mixing equal weight of all samples.

PQ powder of 100 mg was accurately weighed into the Eppendorf tubes. Next, 1.5 mL extraction solution of methanol/water (1:1, *v*/*v*) was added. The mixture was vortexed for 5 min by the vortex oscillator (Digital Vortex-Genie 2, Scientific Industries, Bohemia, NY, USA). Then, the mixture was centrifuged for 10 min and the supernatant was taken out for LC-MS analysis. Three parallel samples were prepared.

### 3.3. LC-MS Analysis

The extract was analyzed by the UPLC system (Water, H-Class, Miford, MA, USA) coupled to a Q-TOF mass spectrometer equipped with an electrospray ionization interface (Bruker Impact II, Bremen, Germany). The LC separation was performed on an Agilent SB-Aq column (2.1 × 100 mm, 1.8 μm) with mobile phase A (0.1% formic acid in water) and mobile phase B (100% acetonitrile). The elution gradient was as follows: 0 min, 5% B; to 2 min, 7% B; to 3 min, 20% B; to 9 min, 24% B, and kept for 4 min; to 16 min, 26% B; to 18 min, 28% B; to 22 min, 34% B, and kept for 8 min; to 34 min, 36% B; to 35 min, 40% B; to 40 min, 50% B; to 50 min, 100% B, and kept for 5 min; to 55.1 min, 5%, and maintained for 5 min. The total run time was 60 min. The flow rate was 0.3 mL/min and column temperature was controlled at 35 °C. The mass data were acquired in the negative mode. The scan was set at a range of 50 to 1500 *m*/*z*. The capillary voltage was set at 3000 v for negative ion mode. The dry flow was set to 8 L/min, the nebulizer pressure was 2.0 bar, and the drying gas temperature was 200 °C. The prepulse storage was 8 μs, the collision RF was 750 Vpp, and the transfer time was 80 μs. The collision energies were set at 40–70 eV.

### 3.4. Data Analysis

MS-DIAL software (version 4.9.0)was used for date deconvolution and peak alignment. A dataset containing *m*/*z*, retention time and peak area was obtained. Principal component analysis (PCA) was performed using Simca 14.0 (Umetrics, Umeå, Sweden). The hierarchical cluster analysis and non-parametric test were performed using MultiExperiment Viewer software (version 4.9, Dana-Farber Cancer Institute, Boston, MA, USA). The select mode for non-parametric test was Wilcoxon and Mann–Whitney test (one factor, two experimental groups).

## 4. Conclusions

A comprehensive investigation was carried out on the ginsenoside identification and transformation of PQ samples with different steaming conditions. In total, 175 ginsenosides were identified, and the sugar chains were annotated based on UHPLC-QTOF-MS. New ginsenosides and isomers were discovered. The steaming process was an effective method to increase the chemical diversity of the ginsenosides. The types and contents of the ginsenosides were found to vary greatly. The content of acylated ginsenosides and protoginsenosides decreased, while the content of the rarest ginsenosides significantly increased after the steaming process. This study can deepen the understanding of the ginsenosides’ conversion in PQ during the steaming process. Since the definite functions of the individual ginsenoside have not been revealed clearly, further research is needed.

## Figures and Tables

**Table 1:** Ginsenosides identified in the steamed PQ samples.

No.	*m*/*z*	t_R_/min	Ion Adducts	Formula	Aglycone	Sugar Chains	Proposed Name	Type
1	793.4388	29.4	[M − H]^−^	C_42_H_66_O_14_	455	*O*-176/*O*-162	Chikusetsusaponin Iva	OA
2	793.4388	33.8	[M − H]^−^	C_42_H_66_O_14_	455	*O*-162/*O*-176	Chikusetsusaponin Iva isomer I	OA
3	793.4388	24.1	[M − H]^−^	C_42_H_66_O_14_	455	*O*-176-162	Zingibroside R1	OA
4	955.4896	22.3	[M − H]^−^	C_48_H_76_O_19_	455	*O*-162-162/*O*-176	Ginsenoside Ro isomer	OA
5	955.4896	21.4	[M − H]^−^	C_48_H_76_O_19_	455	*O*-176-162/*O*-162	Ginsenoside Ro *	OA
6	663.4108	39.8	[M + HCOO]^−^	C_36_H_58_O_8_	455	*O*-162	OA-*O*-glc	OA
7	699.4323	12.2	[M + HCOO]^−^	C_36_H_62_O_10_	491	*O*-162	Pseudoginsenoside Rt4 isomer I	OT
8	699.4323	15.2	[M + HCOO]^−^	C_36_H_62_O_10_	491	*O*-162	Pseudoginsenoside Rt4 isomer II	OT
9	699.4323	11.1	[M + HCOO]^−^	C_36_H_62_O_10_	491	*O*-162	Pseudoginsenoside Rt4	OT
10	785.4693	14.0	[M − H]^−^	C_41_H_70_O_14_	491	*O*-162/*O*-132	Presudoginsenoside FT2	OT
11	825.4634	26.1	[M + HCOO]^−^	C_42_H_68_O_13_	455	*O*-162-162	OA-*O*-glc-glc	OT
12	831.4743	13.8	[M + HCOO]^−^	C_41_H_70_O_14_	491	*O*-162-132	Majonoside R2	OT
13	831.4743	10.4	[M + HCOO]^−^	C_41_H_70_O_14_	491	*O*-162-132	Pseudoginsenoside Rt2	OT
14	845.4900	9.3	[M + HCOO]^−^	C_42_H_72_O_14_	491	*O*-162-146	Pseudoginsenoside F11 isomer III	OT
15	845.4900	11.0	[M + HCOO]^−^	C_42_H_72_O_14_	491	*O*-162-146	Pseudoginsenoside F11 isomer I	OT
16	845.4900	12.0	[M + HCOO]^−^	C_42_H_72_O_14_	491	*O*-162-146	Pseudoginsenoside F11 isomer II	OT
17	845.4900	15.0	[M + HCOO]^−^	C_42_H_72_O_14_	491	*O*-162-146	24(*R*)-Pseudoginsenoside F11	OT
18	845.4900	14.7	[M + HCOO]^−^	C_42_H_72_O_14_	491	*O*-162-146	24(*S*)-Pseudoginsenoside F11 *	OT
19	861.4845	11.7	[M + HCOO]^−^	C_42_H_72_O_15_	491	*O*-162-162	Majonoside R1	OT
20	861.4845	6.4	[M + HCOO]^−^	C_42_H_72_O_15_	491	*O*-162/*O*-162	Majonoside R1 isomer	OT
21	885.4851	17.8	[M − H]^−^	C_45_H_74_O_17_	491	*O*-162-146	Malonyl-Pseudoginsenoside F11	OT
22	887.4991	21.2	[M + HCOO]^−^	C_44_H_74_O_15_	491	*O*-162-146	24(*R*)-Vinaginsenoside R1	OT
23	887.4991	20.6	[M + HCOO]^−^	C_44_H_74_O_15_	491	*O*-162-146	24(*S*)-Vinaginsenoside R1	OT
24	977.5322	7.2	[M + HCOO]^−^	C_47_H_80_O_18_	491	*O*-132/*O*-162-146	OT-*O*-glc-rha-/*O*-xyl/ara	OT
25	815.4793	19.8	[M + HCOO]^−^	C_41_H_70_O_13_	491	*O*-146/*O*-132	OT-*O*-rha-xyl/ara	OT
26	667.4426	41.6	[M + HCOO]^−^	C_36_H_62_O_8_	459	*O*-162	20(*R*)-Ginsenoside Rh2 *	PPD
27	667.4426	40.9	[M + HCOO]^−^	C_36_H_62_O_8_	459	*O*-162	20(*S*)-Ginsenoside Rh2 *	PPD
28	799.4845	37.8	[M + HCOO]^−^	C_41_H_70_O_12_	459	*O*-162-132	20(*S*)-Gypenoside XIII	PPD
29	799.4845	38.6	[M + HCOO]^−^	C_41_H_70_O_12_	459	*O*-162-132	20(*R*)-Gypenoside XIII	PPD
30	829.4943	34.1	[M + HCOO]^−^	C_42_H_72_O_13_	459	*O*-162-162	20(*S*)-Ginsenoside Rg3 *	PPD
31	829.4943	34.6	[M + HCOO]^−^	C_42_H_72_O_13_	459	*O*-162-162	20(*R*)-Ginsenoside Rg3 *	PPD
32	829.4943	37.5	[M + HCOO]^−^	C_42_H_72_O_13_	459	*O*-162-162	Ginsenoside Rg3 isomer II	PPD
33	829.4943	36.2	[M + HCOO]^−^	C_42_H_72_O_13_	459	*O*-162-162	Ginsenoside Rg3 isomer I	PPD
34	829.4943	27.6	[M + HCOO]^−^	C_42_H_72_O_13_	459	*O*-162-162	Ginsenoside Rg3 isomer III	PPD
35	869.489	35.8	[M − H]^−^	C_45_H_74_O_16_	459	*O*-162-162	Malonyl-Ginsenoside Rg3	PPD
36	869.489	37.1	[M − H]^−^	C_45_H_74_O_16_	459	*O*-162-162	Malonyl-Ginsenoside Rg3 isomer I	PPD
37	869.489	37.5	[M − H]^−^	C_45_H_74_O_16_	459	*O*-162-162	Malonyl-Ginsenoside Rg3 isomer II	PPD
38	871.5054	38.0	[M + HCOO]^−^	C_44_H_74_O_14_	459	*O*-162-162	Acetyl-Ginsenoside Rg3	PPD
39	961.5385	30.9	[M + HCOO]^−^	C_47_H_80_O_17_	459	*O*-162-132/*O*-162	Gypenoside IX	PPD
40	961.5385	29.2	[M + HCOO]^−^	C_47_H_80_O_17_	459	*O*-162-132/*O*-162	Notoginsenoside Fe	PPD
41	961.5385	30.3	[M + HCOO]^−^	C_47_H_80_O_17_	459	*O*-162-132/*O*-162	Saponin Ia	PPD
42	961.5385	27.2	[M + HCOO]^−^	C_47_H_80_O_17_	459	*O*-162-162/*O*-132	Vinaginsenoside R17	PPD
43	991.5466	25.2	[M + HCOO]^−^	C_48_H_82_O_18_	459	*O*-162-162/*O*-162	Gypenoside XVII	PPD
44	991.5466	24.7	[M + HCOO]^−^	C_48_H_82_O_18_	459	*O*-162/*O*-162-162	Ginsenoside Rd *	PPD
45	1031.5432	26.0	[M − H]^−^	C_51_H_84_O_21_	459	*O*-162-162/*O*-162	Malonyl-Ginsenoside Rd	PPD
46	1031.5432	27.2	[M − H]^−^	C_51_H_84_0_21_	459	*O*-162-162/*O*-162	Malonyl-Ginsenoside Rd isomer I	PPD
47	1031.5432	25.6	[M − H]^−^	C_51_H_84_0_21_	459	*O*-162-162/*O*-162	Malonyl-Ginsenoside Rd isomer II	PPD
48	1031.5432	28.3	[M − H]^−^	C_51_H_84_O_21_	459	*O*-162-162/*O*-162	Malonyl-Ginsenoside Rd isomer III	PPD
49	1033.5566	30.8	[M + HCOO]^−^	C_50_H_84_O_19_	459	*O*-162-162/*O*-162	Acetyl-Ginsenoside Rd isomer II	PPD
50	1033.5566	34.0	[M + HCOO]^−^	C_50_H_84_O_19_	459	*O*-162-162/*O*-162	Acetyl-Ginsenoside Rd isomer I	PPD
51	1033.5566	29.3	[M + HCOO]^−^	C_50_H_84_O_19_	459	*O*-162-162/*O*-162	Acetyl-Ginsenoside Rd	PPD
52	1107.5945	22.6	[M − H]^−^	C_54_H_92_O_23_	459	*O*-162-162/*O*-162-162	Ginsenoside Rb1 *	PPD
53	1107.5945	22.9	[M − H]^−^	C_54_H_92_O_23_	459	*O*-162-162/*O*-162-162	Ginsenoside Rb1 isomer I	PPD
54	1123.5897	23.3	[M + HCOO]^−^	C_53_H_90_O_22_	459	*O*-162-132/*O*-162-162	Ginsenoside Rb3	PPD
55	1123.5897	23.6	[M + HCOO]^−^	C_53_H_90_O_22_	459	*O*-162-132/*O*-162-162	Ginsenoside Rc	PPD
56	1123.5897	23.9	[M + HCOO]^−^	C_53_H_90_O_22_	459	*O*-162-132/*O*-162-162	Ginsenoside Rb2 *	PPD
57	1137.6053	24.0	[M + HCOO]^−^	C_54_H_92_O_22_	459	*O*-162-162/*O*-162-146	PPD-*O*-glc-glc/*O*-glc-rha	PPD
58	1163.5850	24.4	[M − H]^−^	C_56_H_92_O_25_	459	*O*-162-162/*O*-162-132	Malonyl-Ginsenoside Rb2	PPD
59	1163.5850	23.7	[M − H]^−^	C_56_H_92_O_25_	459	*O*-162-162/*O*-162-132	Malonyl-Ginsenoside Rb3	PPD
60	1163.5850	24.2	[M − H]^−^	C_56_H_92_O_25_	459	*O*-162-162/*O*-162-132	Malonyl-Ginsenoside Rc	PPD
61	1163.5850	24.7	[M − H]^−^	C_56_H_92_O_25_	459	*O*-162-162/*O*-162-132	Malonyl-Ginsenoside Rb2 isomer	PPD
62	1165.5992	27.0	[M + HCOO]^−^	C_55_H_92_O_23_	459	*O*-162-162/*O*-162-132	Acetyl-Ginsenoside Rc	PPD
63	1165.5992	25.9	[M + HCOO]^−^	C_55_H_92_O_23_	459	*O*-162-162/*O*-162-132	Acetyl-Ginsenoside Rb3	PPD
64	1165.5992	30.0	[M + HCOO]^−^	C_55_H_92_O_23_	459	*O*-162-162/*O*-162-132	Acetyl-Ginsenoside Rb2	PPD
65	1193.5959	24.6	[M − H]^−^	C_57_H_94_O_26_	459	*O*-162-162/*O*-162-162	Malonyl-Ginsenoside Rb1 isomer II	PPD
66	1193.5959	23.2	[M − H]^−^	C_57_H_94_O_26_	459	*O*-162-162/*O*-162-162	Malonyl-Ginsenoside Rb1	PPD
67	1193.5959	24.1	[M − H]^−^	C_57_H_94_O_26_	459	*O*-162-162/*O*-162-162	Malonyl-Ginsenoside Rb1 isomer I	PPD
68	1195.6107	25.0	[M + HCOO]^−^	C_56_H_94_O_24_	459	*O*-162-162/*O*-162-162	Acetyl-Ginsenoside Rb1	PPD
69	1195.6107	25.7	[M + HCOO]^−^	C_56_H_94_O_24_	459	*O*-162-162/*O*-162-162	Acetyl-Ginsenoside Rb1 isomer I	PPD
70	1195.6107	27.5	[M + HCOO]^−^	C_56_H_94_O_24_	459	*O*-162-162/*O*-162-162	Acetyl-Ginsenoside Rb1 isomer II	PPD
71	975.5534	30.4	[M + HCOO]^−^	C_48_H_82_O_17_	459	*O*-162-162/*O*-146	PPD-*O*-glc-glc/*O*-rha	PPD
72	975.5534	31.3	[M + HCOO]^−^	C_48_H_82_O_17_	459	*O*-162-162/*O*-146	PPD-*O*-glc-glc/*O*-rha isomer I	PPD
73	975.5534	29.9	[M + HCOO]^−^	C_48_H_82_O_17_	459	*O*-162-162/*O*-146	PPD-*O*-glc-glc/*O*-rha isomer II	PPD
74	825.4975	36.0	[M + HCOO]^−^	C_43_H_72_O_12_	459	*O*-162/*O*-162	Acetyl-PPD-*O*-glc/*O*-glc	PPD
75	631.3842	38.3	[M − H]^−^	C_36_H_56_O_9_	475	*O*-176	PPT-*O*-glcA	PPT
76	683.4366	15.5	[M + HCOO]^−^	C_36_H_62_O_9_	475	*O*-162	Chikusetsusaponin L10	PPT
77	683.4366	17.0	[M + HCOO]^−^	C_36_H_62_O_9_	475	*O*-162	Ginsenoside Rh1 isomer	PPT
78	683.4366	19.3	[M + HCOO]^−^	C_36_H_62_O_9_	475	*O*-162	20(*S*)-Ginsenoside Rh1 *	PPT
79	683.4366	20.7	[M + HCOO]^−^	C_36_H_62_O_9_	475	*O*-162	20(*R*)-Ginsenoside Rh1	PPT
80	683.4366	22.9	[M + HCOO]^−^	C_36_H_62_O_9_	475	*O*-162	Ginsenoside F1	PPT
81	815.4793	20.4	[M + HCOO]^−^	C_41_H_70_O_13_	475	*O*-162-132	Ginsenoside F5	PPT
82	815.4793	19.1	[M + HCOO]^−^	C_41_H_70_O_13_	475	*O*-162-132	Ginsenoside F3	PPT
83	815.4793	17.4	[M + HCOO]^−^	C_41_H_70_O_13_	475	*O*-162-132	Notoginsenoside R2	PPT
84	815.4793	11.5	[M + HCOO]^−^	C_41_H_70_O_13_	475	*O*-162/*O*-132	PPT-*O*-xyl/ara/*O*-glc	PPT
85	815.4793	18.1	[M + HCOO]^−^	C_41_H_70_O_13_	475	*O*-162/*O*-132	PPT-*O*-xyl/ara/*O*-glc isomer	PPT
86	829.4943	19.3	[M + HCOO]^−^	C_42_H_72_O_13_	475	*O*-162-146	20(*S*)-Ginsenoside Rg2 *	PPT
87	829.4943	20.0	[M + HCOO]^−^	C_42_H_72_O_13_	475	*O*-162-146	20(*R*)-Ginsenoside Rg2 *	PPT
88	829.4943	15.5	[M + HCOO]^−^	C_42_H_72_O_13_	475	*O*-162-146	Ginsenoside Rg2 isomer I	PPT
89	829.4943	16.3	[M + HCOO]^−^	C_42_H_72_O_13_	475	*O*-162-146	Ginsenoside Rg2 isomer II	PPT
90	845.4913	8.8	[M + HCOO]^−^	C_42_H_72_O_14_	475	*O*-162/*O*-162	Ginsenoside Rg1 *	PPT
91	845.4913	16.6	[M + HCOO]^−^	C_42_H_72_O_14_	475	*O*-162-162	Ginsenoside Rf	PPT
92	845.4913	21.9	[M + HCOO]^−^	C_42_H_72_O_14_	475	*O*-162/*O*-162	Ginsenoside La	PPT
93	869.4890	21.3	[M − H]^−^	C_45_H_74_O_16_	475	*O*-162-146	Malonyl-Ginsenoside Rg2	PPT
94	885.4851	10.5	[M − H]^−^	C_45_H_74_O_17_	475	*O*-162-162	Malonyl-Ginsenoside Rf	PPT
95	887.4991	13.7	[M + HCOO]^−^	C_44_H_74_O_15_	475	*O*-162-162	Acetyl-20(*R*)-Ginsenoside Rg2	PPT
96	887.4991	13	[M + HCOO]^−^	C_44_H_74_O_15_	475	*O*-162/*O*-162	Acetyl-20(*S*)-Ginsenoside Rg1	PPT
97	887.4991	14.4	[M + HCOO]^−^	C_44_H_74_O_15_	475	*O*-162/*O*-162	Acetyl-20(*R*)-Ginsenoside Rg1	PPT
98	961.5385	10.4	[M + HCOO]^−^	C_47_H_80_O_17_	475	*O*-162-146/*O*-132	Cyclofoetoside A isomer	PPT
99	961.5385	11.7	[M + HCOO]^−^	C_47_H_80_O_17_	475	*O*-162-146/*O*-132	Cyclofoetoside A	PPT
100	977.5322	17.8	[M + HCOO]^−^	C_47_H_80_O_18_	475	*O*-162/*O*-162-132	Quinquenoside L3	PPT
101	977.5322	7.7	[M + HCOO]^−^	C_47_H_80_O_18_	475	*O*-162-132/*O*-162	Ginsenoside Re4	PPT
102	977.5322	8.1	[M + HCOO]^−^	C_47_H_80_O_18_	475	*O*-162-132/*O*-162	20(*S*)-Quinquenoside L17	PPT
103	977.5322	8.4	[M + HCOO]^−^	C_47_H_80_O_18_	475	*O*-162-132/*O*-162	20(*R*)-Quinquenoside L17	PPT
104	991.5466	8.7	[M + HCOO]^−^	C_48_H_82_O_18_	475	*O*-162-146/*O*-162	Ginsenoside Re isomer	PPT
105	991.5466	27.5	[M + HCOO]^−^	C_48_H_82_O_18_	475	*O*-162/*O*-162-162	Chikusetsusaponin FK1	PPT
106	991.5466	9.5	[M + HCOO]^−^	C_48_H_82_O_18_	475	*O*-162-146/*O*-162	Ginsenoside Re *	PPT
107	1007.5416	6.9	[M + HCOO]^−^	C_48_H_82_O_19_	475	*O*-162/*O*-162-162	Ginsenoside Re1	PPT
108	1007.5416	13.4	[M + HCOO]^−^	C_48_H_82_O_19_	475	*O*-162/*O*-162-162	Ginsenoside Re2	PPT
109	1007.5416	7.4	[M + HCOO]^−^	C_48_H_82_O_19_	475	*O*-162/*O*-162-162	Ginsenoside Re3	PPT
110	1031.5432	10.7	[M − H]^−^	C_51_H_84_0_21_	475	*O*-162-146/*O*-162	Malonyl-Ginsenoside Re	PPT
111	1031.5432	11.4	[M − H]^−^	C_51_H_84_0_21_	475	*O*-162-146/*O*-162	Malonyl-Ginsenoside Re isomer I	PPT
112	1031.5432	12.0	[M − H]^−^	C_51_H_84_0_21_	475	*O*-162-146/*O*-162	Malonyl-Ginsenoside Re isomer II	PPT
113	1031.5432	12.5	[M − H]^−^	C_51_H_84_0_21_	475	*O*-162-146/*O*-162	Malonyl-Ginsenoside Re isomer III	PPT
114	1033.5566	15.4	[M + HCOO]^−^	C_50_H_84_O_19_	475	*O*-162-146/*O*-162	Acetyl-Ginsenoside Re	PPT
115	1033.5566	14.0	[M + HCOO]^−^	C_50_H_84_O_19_	475	*O*-162-146/*O*-162	Acetyl-Ginsenoside Re isomer	PPT
116	1139.5848	11.1	[M + HCOO]^−^	C_53_H_90_O_23_	475	*O*-162-162/*O*-162-132	Floralginsenoside P	PPT
117	1123.5897	7.9	[M + HCOO]^−^	C_53_H_90_O_22_	475	*O*-162-132/*O*-162-146	Floralquinquenoside E	PPT
118	799.4845	23.4	[M + HCOO]^−^	C_41_H_70_O_12_	475	*O*-146-132	PPT-*O*-rha-xyl/ara	PPT
119	975.5534	15.3	[M + HCOO]^−^	C_48_H_82_O_17_	475	*O*-162-146/*O*-146	PPT-*O*-glc-rha/*O*-rha **	PPT
120	665.4265	28.3	[M + HCOO]^−^	C_36_H_60_O_8_	457	*O*-162	Ginsenoside Rh4	OTHER
121	665.4265	27.0	[M + HCOO]^−^	C_36_H_60_O_8_	457	*O*-162	Ginsenoside Pk3	OTHER
122	665.4265	26.7	[M + HCOO]^−^	C_36_H_60_O_8_	457	*O*-162	Ginsenoside Pk3 isomer	OTHER
123	665.4265	40.3	[M + HCOO]^−^	C_36_H_60_O_8_	457	*O*-162	Ginsenoside Rh16	OTHER
124	665.4265	42.0	[M + HCOO]^−^	C_36_H_60_O_8_	457	*O*-162	Ginsenoside Rh16 isomer	OTHER
125	701.4475	8.6	[M − H]^−^	C_36_H_64_O_10_	493	*O*-162	20(*S*)-Ginsenoside Rh12	OTHER
126	701.4475	9.3	[M − H]^−^	C_36_H_64_O_10_	493	*O*-162	20(*R*)-Ginsenoside Rh12	OTHER
127	703.4207	17.2	[M + HCOO]^−^	C_35_H_62_O_11_	495	*O*-162	Floralginsenoside Tb	OTHER
128	717.4421	6.6	[M + HCOO]^−^	C_36_H_64_O_11_	509	*O*-162	Dammarane-3,6,12,24,25-pentol, 20-(β-d-glucopyranosyloxy)-, (3β,6β,12β)-(ACI) isomer	OTHER
129	717.4421	7.8	[M + HCOO]^−^	C_36_H_64_O_11_	509	*O*-162	Dammarane-3,6,12,24,25-pentol, 20-(β-d-glucopyranosyloxy)-, (3β,6β,12β)-(ACI)	OTHER
130	781.4734	40.7	[M + HCOO]^−^	C_41_H_68_O_11_	441	*O*-162-132	Notoginsenoside ST11	OTHER
131	811.4839	25.4	[M + HCOO]^−^	C_42_H_70_O_12_	457	*O*-162-146	Ginsenoside Rg6 isomer I	OTHER
132	811.4839	25.8	[M + HCOO]^−^	C_42_H_70_O_12_	457	*O*-162-146	Ginsenoside Rg6 isomer II	OTHER
133	811.4839	26.4	[M + HCOO]^−^	C_42_H_70_O_12_	457	*O*-162-146	Ginsenoside Rg6	OTHER
134	811.4839	38.6	[M + HCOO]^−^	C_42_H_70_O_12_	441	*O*-162-162	Ginsenoside Rg5 isomer I	OTHER
135	811.4839	39.5	[M + HCOO]^−^	C_42_H_70_O_12_	441	*O*-162-162	Ginsenoside Rg5 *	OTHER
136	811.4839	39.0	[M + HCOO]^−^	C_42_H_70_O_12_	441	*O*-162-162	Ginsenoside Rg5 isomer II	OTHER
137	827.4783	38.4	[M + HCOO]^−^	C_42_H_70_O_13_	457	*O*-162-162	20(*R*)-5,6-Didehydroginsenoside Rg3	OTHER
138	827.4783	32.2	[M + HCOO]^−^	C_42_H_70_O_13_	457	*O*-162-162	20(*S*)-5,6-Didehydroginsenoside Rg3	OTHER
139	827.4783	28.0	[M + HCOO]^−^	C_42_H_70_O_13_	457	*O*-162-162	Ginsenoside Rh15	OTHER
140	847.5047	8.6	[M + HCOO]^−^	C_42_H_74_O_14_	493	*O*-162-146	20(*S*)-25-OH-Ginsenoside Rg2	OTHER
141	847.5047	23.3	[M + HCOO]^−^	C_42_H_74_O_14_	477	*O*-162-162	20(*S*)-25-OH-Ginsenoside Rg3	OTHER
142	847.5047	24.0	[M + HCOO]^−^	C_42_H_74_O_14_	477	*O*-162-162	20(*R*)-25-OH-Ginsenoside Rg3	OTHER
143	849.4843	6.5	[M + HCOO]^−^	C_41_H_72_O_15_	509	*O*-162/*O*-132	β-d-Glucopyranoside, (3β,12β)-3,12,24,25-tetrahydroxy-20-(d-xylopyranosyloxy)dammaran-6-yl (ACI)	OTHER
144	853.4949	42.0	[M + HCOO]^−^	C_44_H_72_O_13_	441	*O*-162-162	Ginsenoside Rs4	OTHER
145	863.501	6.7	[M + HCOO]^−^	C_42_H_74_O_15_	509	*O*-162-146	Quinquenoside L9	OTHER
146	975.5534	24.3	[M + HCOO]^−^	C_48_H_82_O_17_	443	*O*-162-162/*O*-162	Vinaginsenoside R3	OTHER
147	989.5324	24.0	[M + HCOO]^−^	C_48_H_80_O_18_	457	*O*-162-162/*O*-162	5,6-Didehydroginsenoside Rd	OTHER
148	989.5324	13.0	[M + HCOO]^−^	C_48_H_80_O_18_	473	*O*-162/*O*-162-146	Ginsenoside Rh18	OTHER
149	989.5324	20.8	[M + HCOO]^−^	C_48_H_80_O_18_	457	*O*-162-162/*O*-162	Quinquenoside L1	OTHER
150	1005.527	10.7	[M + HCOO]^−^	C_48_H_80_O_19_	473	*O*-162-162/*O*-162	Vinaginsenoside R20	OTHER
151	1007.5416	6.3	[M + HCOO]^−^	C_48_H_82_O_19_	491	*O*-162-146/*O*-146	Majoroside F5	OTHER
152	1007.5416	10.2	[M + HCOO]^−^	C_48_H_82_O_19_	491	*O*-162/*O*-162-146	Majoroside F6	OTHER
153	1009.559	5.7	[M + HCOO]^−^	C_48_H_84_O_19_	493	*O*-162/*O*-162-146	β-d-Glucopyranoside, (3β,6α,12β)-20-(β-d-glucopyranosyloxy)-3,12,25-trihydroxydammaran-6-yl 2-*O*-(6-deoxy-α-L-mannopyranosyl)- (ACI)	OTHER
154	1025.5541	10.6	[M + HCOO]^−^	C_48_H_84_O_20_	493	*O*-162-162/*O*-162	Vinaginsenoside R13 isomer	OTHER
155	1025.5541	12.0	[M + HCOO]^−^	C_48_H_84_O_20_	493	*O*-162/*O*-162-162	Vinaginsenoside R13	OTHER
156	1137.6053	21.9	[M + HCOO]^−^	C_54_H_92_O_22_	443	*O*-162-162/*O*-162-162	Notoginsenoside I	OTHER
157	1151.5848	22.0	[M + HCOO]^−^	C_54_H_90_O_23_	457	*O*-162-162/*O*-162-162	5,6-Didehydroginsenoside Rb1	OTHER
158	1167.5913	8.5	[M + HCOO]^−^	C_54_H_90_O_24_	473	*O*-162-162/*O*-162-162	Notoginsenoside B	OTHER
159	1169.5943	10.2	[M + HCOO]^−^	C_54_H_92_0_24_	475	*O*-162-162/*O*-162-162	Koryoginsenoside R2	OTHER
160	1169.5943	12.6	[M + HCOO]^−^	C_54_H_92_O_24_	475	*O*-162-162/*O*-162-162	Notoginsenoside A	OTHER
161	781.4734	41.3	[M + HCOO]^−^	C_41_H_68_O_11_	441	*O*-162-132	Notoginsenoside ST11 isomer	OTHER
162	843.4948	19.7	[M + HCOO]^−^	C_42_H_70_O_14_	473	*O*-162-162	11-Oxomogroside II A1	OTHER
163	973.5373	16.2	[M + HCOO]^−^	C_48_H_80_O_17_	473	*O*-162-146/*O*-146	(3β,16β,22α)-28-[(6-Deoxy-α-L-mannopyranosyl)oxy]-16,22-dihydroxyolean-12-en-3-yl 6-deoxy-3-*O*-β-d-glucopyranosyl-α-L-mannopyranoside	OTHER
164	973.5373	15.0	[M + HCOO]^−^	C_48_H_80_O_17_	473	*O*-162-146/*O*-146	(3β,16β,22α)-28-[(6-Deoxy-α-l-mannopyranosyl)oxy]-16,22-dihydroxyolean-12-en-3-yl 6-deoxy-3-*O*-β-d-glucopyranosyl-α-l-mannopyranoside isomer	OTHER
165	1009.5575	19.1	[M + HCOO]^−^	C_48_H_84_O_19_	477	*O*-162-162/*O*-162	(3β,12β)-20-(β-d-Glucopyranosyloxy)-12,25-dihydroxydammaran-3-yl 2-*O*-β-d-glucopyranosyl-β-d-glucopyranoside	OTHER
166	1025.5541	5.1	[M + HCOO]^−^	C_48_H_84_O_20_	509	*O*-162/*O*-162-146	(3β,12β)-20-(β-d-Glucopyranosyloxy)-3,12,24,25-tetrahydroxydammaran-6-yl 2-*O*-(6-deoxy-α-l-β-d-mannopyranosyl)-β-d-glucopyranoside	OTHER
167	803.4426	34.1	[M + HCOO]^−^	C_39_H_66_O_14_	433	*O*-162-162	PQ-ginsenoside A **	OTHER
168	803.4426	36.0	[M + HCOO]^−^	C_39_H_66_O_14_	433	*O*-162-162	PQ-ginsenoside A isomer **	OTHER
169	803.4426	19.3	[M + HCOO]^−^	C_39_H_66_O_14_	449	*O*-162-146	PQ-ginsenoside B **	OTHER
170	785.4322	26.4	[M + HCOO]^−^	C_40_H_66_O_15_	431	*O*-162-146	PQ-ginsenoside C **	OTHER
171	785.4322	39.0	[M + HCOO]^−^	C_40_H_66_O_15_	415	*O*-162-162	PQ-ginsenoside D **	OTHER
172	827.4438	41.7	[M + HCOO]^−^	C_41_H_66_O_14_	415	*O*-162-162	Acetyl-PQ-ginsenoside D **	OTHER
173	827.4438	41.3	[M + HCOO]^−^	C_41_H_66_O_14_	415	*O*-162-162	Acetyl-PQ-ginsenoside D isomer I **	OTHER
174	827.4438	42.0	[M + HCOO]^−^	C_41_H_66_O_14_	415	*O*-162-162	Acetyl-PQ-ginsenoside D isomer II **	OTHER
175	845.4536	40.0	[M + HCOO]^−^	C_41_H_68_O_15_	433	*O*-162-162	Acetyl-PQ-ginsenoside A **	OTHER

* represents ginsenosides validated with standards. ** represents new ginsenosides.

## Data Availability

Data are contained within the article and Supplementary Materials.

## Supplementary Materials

The following supporting information can be downloaded at: https://www.mdpi.com/article/10.3390/molecules29030623/s1, Figure S1: The peak number and accumulated peak area with different RSD ranges for the repeatability (A), intra-day (B), and inter-day (C) precision. Figure S2: Heat map of differential ginsenosides in PQ samples with different steaming time. A, PPD-type. B, PPT-type. C, OA-type. D, OT-type. E, Other type. Figure S3: The deduced transformation pathways of PPD-type ginsenosides. Figure S4: The deduced transformation pathways of PPT-type ginsenosides. Figure S5: The deduced transformation pathways of OA-type ginsenosides (A) and OT-type ginsenosides (B). Table S1: The *p* and ratio of differential ginsenosides in the samples with different steaming times. Table S2: The *p* and ratio of differential ginsenosides in the samples with different steaming temperatures.
