# Supplementary material for: Comprehensive Investigation of Ginsenosides in the Steamed Panax quinquefolius with Different Processing Conditions Using LC-MS
Source: Molecules. 2024 Jan 28;29(3):623. doi: 10.3390/molecules29030623 (PMC10856252; doi:10.3390/molecules29030623)
Supplement: Supplementary file 1 [file molecules-29-00623-s001.zip › molecules-2828833-supplementary.pdf]

Comprehensive investigation of ginsenosides in the steamed *Panax quinquefolius* with different processing conditions using LC-MS

Jiali Fan[1,2], Feng Liu [1,2], Wenhua Ji[1,2], Xiao Wang[1,2], Lili Li[1,2*]

1 Key Laboratory for Applied Technology of Sophisticated Analytical Instruments of Shandong Province, Shandong Analysis and Test Center, Qilu University of Technology (Shandong Academy of Sciences), Jinan, 250014, China

2 School of Pharmaceutical Sciences, Qilu University of Technology (Shandong Academy of Sciences), Jinan, 250014, China

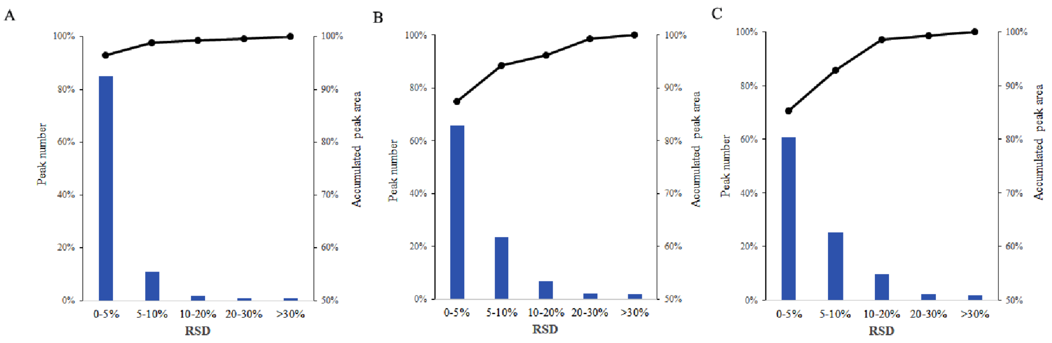

Figure S1. The peak number and accumulated peak area with different RSD ranges for the repeatability (A), intra-day (B), and inter-day (C) precision.

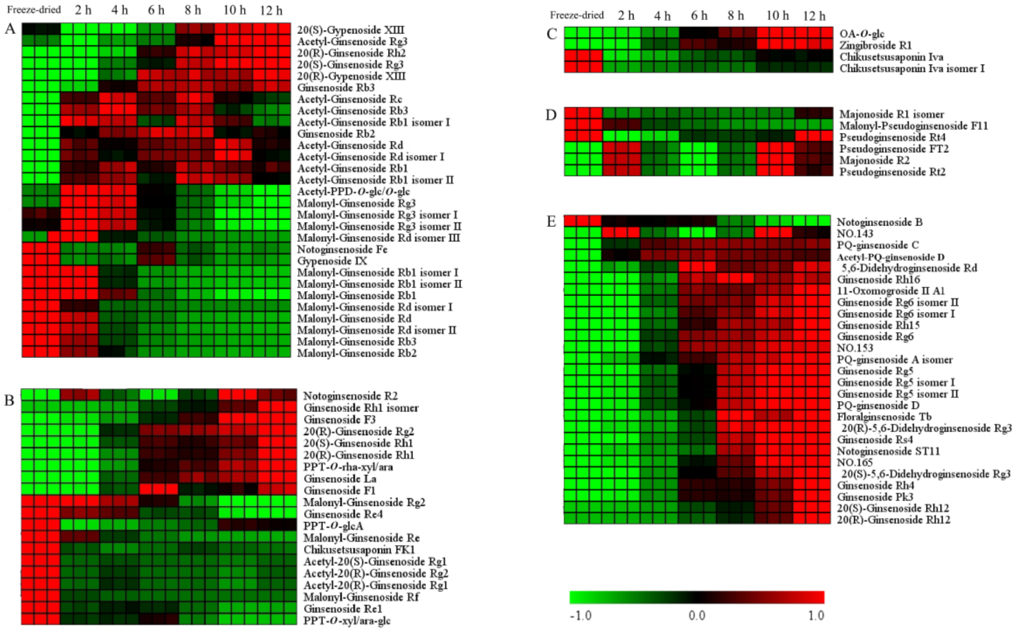

Figure S2. Heat map of differential ginsenosides in PQ samples with different steaming time. A, PPD-type. B, PPT-type. C, OA-type. D, OT-type. E, Other-type. No. 143 represents (3β,16β,22α)-28-[(6-Deoxy-α-L-mannopyranosyl)oxy]-16,22-dihydroxyolean-12-en-3-yl 6-deoxy-3-O-β-D-glucopyranosyl-α-L-mannopyranoside. No. 153 represents β-D-Glucopyranoside,(3β,6α,12β)-20-(β-D-glucopyranosyloxy)-3,12,25-trihydroxydammaran-6-yl 2-O-(6-deoxy-α-L-mannopyranosyl)-(ACI). No. 165 represents (3β,12β)-20-(β-D-Glucopyranosyloxy)-12,25-dihydroxydammaran-3-yl, 2-O-β-D-glucopyranosyl-β-D-glucopyranoside.

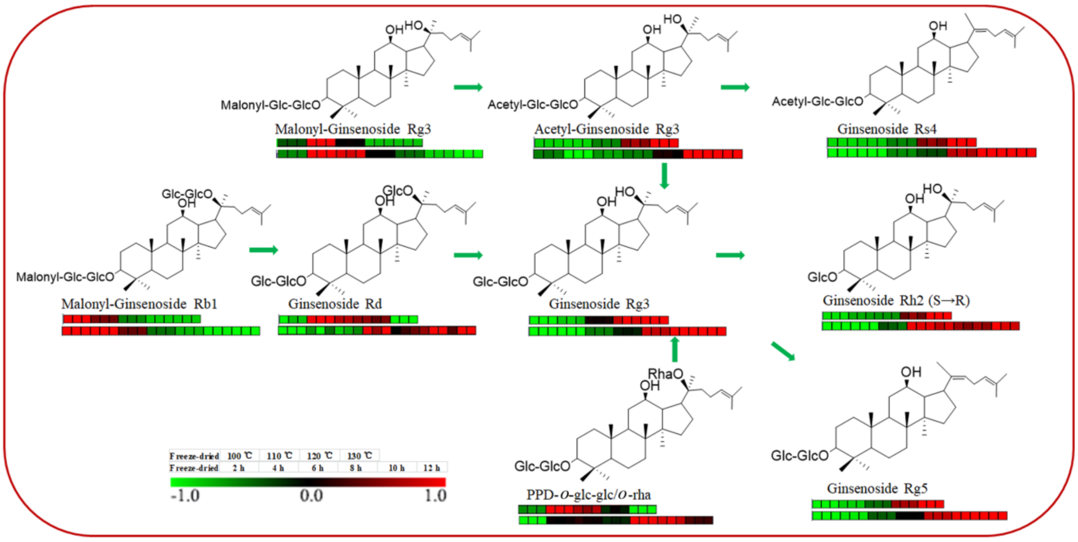

Figure S3. The deduced transformation pathways of PPD-type ginsenosides.

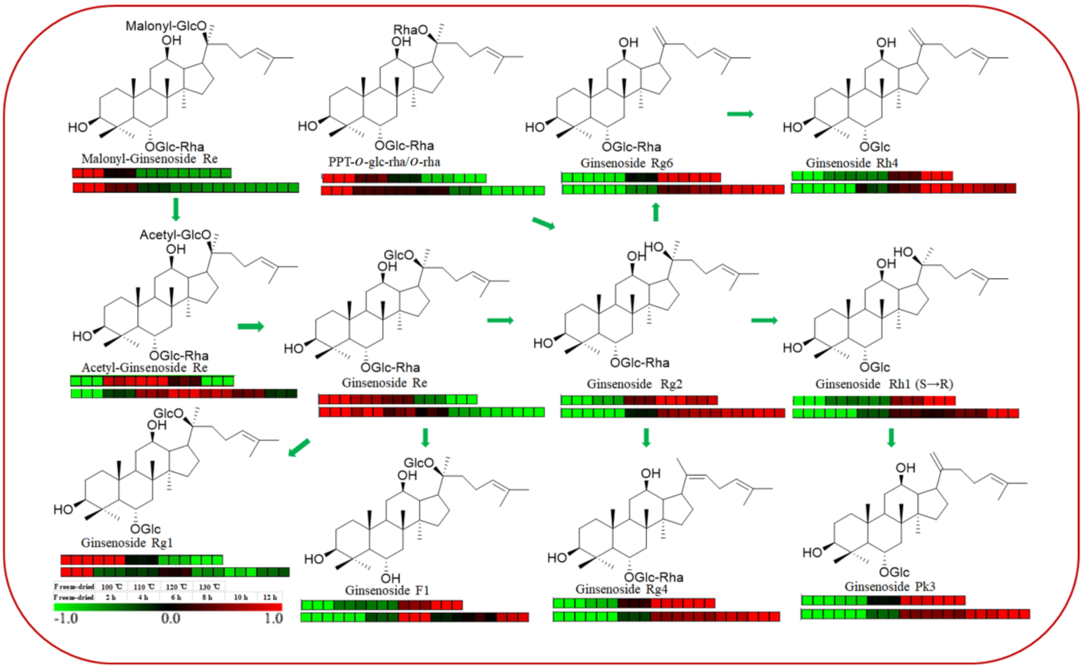

Figure S4. The deduced transformation pathways of PPT-type ginsenosides.

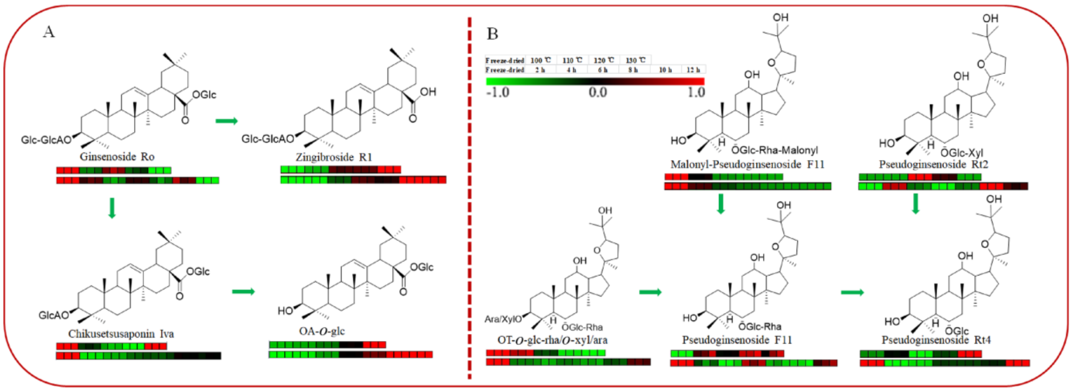

Figure S5. The deduced transformation pathways of OA-type ginsenosides (A) and OT-type ginsenosides (B).
